# Germ cells of the centipede *Strigamia maritima* are specified early in embryonic development

**DOI:** 10.1016/j.ydbio.2014.06.003

**Published:** 2014-08-15

**Authors:** Jack E. Green, Michael Akam

**Affiliations:** Laboratory for Development and Evolution, Department of Zoology, University of Cambridge, Downing Street, Cambridge, CB2 3EJ, UK

**Keywords:** Germ line specification, Vasa, Nanos, Blastopore, Myriapod, Evolution

## Abstract

We provide the first systematic description of germ cell development with molecular markers in a myriapod, the centipede *Strigamia maritima*. By examining the expression of *Strigamia vasa* and *nanos* orthologues, we find that the primordial germ cells are specified from at least the blastoderm stage. This is a much earlier embryonic stage than previously described for centipedes, or any other member of the Myriapoda. Using these genes as markers, and taking advantage of the developmental synchrony of *Strigamia* embryos within single clutches, we are able to track the development of the germ cells throughout embryogenesis. We find that the germ cells accumulate at the blastopore; that the cells do not internalize through the hindgut, but rather through the closing blastopore; and that the cells undergo a long-range migration to the embryonic gonad. This is the first evidence for primordial germ cells displaying these behaviours in any myriapod. The myriapods are a phylogenetically important group in the arthropod radiation for which relatively little developmental data is currently available. Our study provides valuable comparative data that complements the growing number of studies in insects, crustaceans and chelicerates, and is important for the correct reconstruction of ancestral states and a fuller understanding of how germ cell development has evolved in different arthropod lineages.

## Highlights

• First modern description of germ cell development in a myriapod, using molecular markers.
• Germ cells are specified from at least the blastoderm stage in the centipede *Strigamia maritima*.
• This is a much earlier embryonic stage than previously described for any other myriapod.
• The germ cells do not internalize through the hindgut, but through the closing blastopore.
• The germ cells undergo a long-range migration to the embryonic gonad.

## Introduction

The germ cells of an organism are the only cell type that contributes genetically to the next generation. Across the Metazoa, organisms segregate the germ line from the soma, either during embryonic development or in early larval life (reviewed in Extavour and Akam, 2003). The first cells that will give rise exclusively to the germ line are called the primordial germ cells (PGCs). The mechanism of germ line specification varies across taxa and can be viewed as a continuum characterized by two extremes. At one end is a class of mechanisms we call ‘cytoplasmic’. These are characterized by the production of a special region of cytoplasm, either in the unfertilized egg or after fertilization in the early embryo, which is only inherited by a subset of embryonic cells that become the germ cells. This specialized cytoplasm is called germ plasm, and contains localized factors necessary and sufficient for germ line specification. At the opposite extreme is a class of mechanisms that we call ‘induction’. In this class, the germ line is specified by signals sent from neighbouring cells. Evidence now available for mice and for the insect, *Gryllus bimaculatus*, shows that BMP signalling plays a role in induction (Donoughe et al., 2014; Lawson et al., 1999; Ying et al., 2000; Ying and Zhao, 2001).

In three of the four myriapod groups, classical literature describes a late origin of the PGCs from the coelomic pouches of the mesoderm (millipedes, symphylans and pauropods; Dohle, 1964; Tiegs, 1940, 1947). In the fourth myriapod group, the centipedes, classical studies on two species of *Scolopendra* identify a population of cells in the late embryonic/early post-embryonic gonad with distinctive cytological characteristics (larger nuclei and more abundant cytoplasm) that gives rise to the gametes, consistent with its identity as the centipede PGCs (Heymons, 1901). However, Heymons also reports an accumulation of cells earlier in development, at the posterior end of the embryonic rudiment, which later migrate anteriorly and become enclosed within the gonadal epithelium; and he identifies these earlier cells as the PGCs (Heymons, 1901). However, he notes that these cells are not distinguishable morphologically as PGCs until they reach the gonad, and provides little or no evidence for this description in the accompanying figures. Given the ambiguity, previous surveys of germ cell development have concluded that centipede PGCs arise late in embryogenesis from gonadal mesoderm (Extavour and Akam, 2003; Nieuwkoop and Sutasurya, 1981; but not in Johannsen and Butt, 1941). This highlights a general limitation of studies based on recognizing the germ cells cytologically. This limitation means it is not always possible to distinguish a late segregation of germ cells from merely a late acquisition of the distinctive cytological properties. The use of conserved molecular markers may circumvent this problem, and can give a more accurate estimate of the timing of PGC segregation (Tsunekawa et al., 2000; Wu et al., 2011; Yoon et al., 1997).

There are a growing number of studies on insects and crustaceans that are applying molecular techniques to elucidate the timing and mechanism of specification of the PGCs in these taxa (Calvo et al., 2005; Chang et al., 2002, 2006; Dearden, 2006; Ewen-Campen et al., 2013a, 2013b; Extavour, 2005; Gerberding et al., 2002; Gupta and Extavour, 2013; Khila and Abouheif, 2010; Lynch and Desplan, 2010; Nakao, 1999; Nakao et al., 2006; Oezhan-Kizil et al., 2009; Schroder, 2006). The myriapods are an ancient lineage of arthropods, and the living outgroup to the clade containing insects and all crustaceans (Rota-Stabelli et al., 2011). They are therefore phylogenetically well placed to determine ancestral states and the polarity of evolutionary change within the whole group of mandibulate arthropods. However, to our knowledge, at present there are no studies of germ cell development using molecular markers in any myriapod.

In this study we set out to establish, during embryogenesis of the centipede *Strigamia maritima*, when and where the germ cells first appear, and how they subsequently develop. We aimed to shed light on the mechanism of germ line specification in *Strigamia*, and to find evidence that would distinguish between a cytoplasmic and an inductive mode of germ line specification.

We use *Strigamia* orthologues of two conserved molecular markers of PGCs, *vasa* and *nanos*, to show conclusively that the PGCs are specified at least as early as the blastoderm stage, a much earlier embryonic stage than currently believed. This resolves an ambiguity in the older literature. Furthermore, we show that the PGCs accumulate at the blastopore; that they internalize through the closing blastopore, and not through the hindgut; and that they undergo a long-range migration to the embryonic gonad. Finally, we find a surprising localization of maternal Vasa protein within the germinal vesicle of developing oocytes, and suggest that this might act as a mechanism for localizing Vasa protein asymmetrically in early embryos.

## Methods

### Identification and cloning of germ line markers

A genome and adult and embryonic transcriptomes for *S. maritima* (genome release Smar_1.0) are available at http://www.ncbi.nlm.nih.gov/assembly/322118/. An annotated gene set is provided at EnsemblMetazoa (http://metazoa.ensembl.org/Strigamia_maritima/Info/Index) (Chipman et al., submitted). Cassandra Extavour and colleagues identified a set of 31 genes orthologous (one-to-one or one-to-many) to genes known to have roles in germ line specification or differentiation in other species during annotation of the *Strigamia* genome (Chipman et al., submitted). The names and Ensembl IDs of this set are provided in Supplementary Table 1. We designed gene-specific primers for 8 of these putative germ line markers and amplified PCR products from embryonic cDNA. The amplified fragments were cloned into pGEM-T Easy vector (Promega). The names, Ensembl IDs and primer sequences used for this set of 8 genes are provided in Supplementary Table 1. We screened these 8 possible germ line markers by examining their expression in stage 5 *Strigamia* embryos using in situ hybridization. Stage 5 was selected because the germ cells are cytologically differentiated (large round nuclei with diffuse chromatin) and easy to recognize at this stage. Of these 8 candidates, only the *Strigamia* orthologue of *vasa,* and one of the two *nanos* orthologues*, nanos2*, showed unambiguous staining of the putative PGCs.

### Embryo collection, fixation and staging

Embryos were collected in the field from a population near Brora, Scotland and fixed as described previously (Brena and Akam, 2012). Accurate developmental series were obtained by examining the dynamics of a gene expression pattern, or a particular developmental process, within single clutches of *Strigamia* eggs. We took advantage of the degree of developmental synchrony within a clutch to obtain a close time series of embryos, which were placed in developmental order according to morphological criteria in Brena and Akam (2012), or using independent molecular markers, as indicated below.

### Collection of adult females, ovary preparation and fixation

Adult female centipedes were collected at two different sites in the UK and maintained in the laboratory at 10–12 °C for several months. Females were collected from Brora, Scotland in June 2013 and from Brownsea Island, Poole, Dorset in October 2013. Ovaries were dissected from adult females in a solution of phosphate-buffered saline (1× PBS) and 10 mM MgCl_2_ (anaesthetic). Isolated ovaries were fixed immediately in 4% formaldehyde in 1× PBS for 25 min at room temperature. After fixation, ovaries were washed 3 times for 5 min each in 1× PBS and then transferred step-wise into methanol (25:50:75:100%), and stored at −20 °C.

### RNA extraction and RT-PCR

For the RT-PCR experiment, RNA was extracted from pools of embryos from single clutches. Live eggs were returned to the lab on moist Petri dishes, as described in Brena and Akam (2012). To determine the stage of the clutches, two test embryos were removed from each clutch, fixed, DAPI stained to reveal nuclei and staged as in Brena and Akam (2012). The remaining eggs were snap frozen in liquid nitrogen and stored at −80 °C. RNA was extracted from the frozen samples using the RNeasy Mini Kit (Qiagen). Embryos were lysed and homogenized in buffer RLT according to manufacturer’s instructions, except for RNA extractions from clutches at embryonic stages 1 and 2, to which 10 μg of poly-A carrier RNA (Qiagen) was added to the lysate before proceeding with the rest of the protocol. Residual genomic DNA contamination was removed by including the on-column DNase digestion step with the RNase-Free DNase Set in the manufacturer’s protocol (Qiagen). RNA was eluted in 50 μl of RNase-free water. cDNA was synthesized using Expand Reverse Transcriptase (Roche) primed with random hexanucleotides (Roche), according to manufacturer’s instructions (4 μl RNA in 20 μl reaction volume for all samples). Fragments were amplified by PCR (2 μl of cDNA prep in 25 μl PCR) using ThermoPrime Plus DNA polymerase (Thermo Scientific). The product of cDNA synthesis reaction in which no RNA had been added to the reaction was used as a no template control. Primer sequences used to detect *nanos2*, *vasa*, *bra1*, *en* and *tubulin* are provided in Supplementary Table 2. All primers were designed such that the amplified products crossed exon-intron boundaries. PCR products were visualized on 1% agarose gel with 0.1 ng/μl ethidium bromide. PCRs were run under the following conditions: an initial denaturation step of 95 °C for 8 min; followed by 35 cycles of 95 °C for 30 s, 56 °C for 30 s, 72 °C for 1 min; and finally an extension step of 72 °C for 4 min.

### in situ hybridization on embryos and adult ovaries

in situ hybridization reactions were carried out on whole mount embryos as described previously (Chipman et al., 2004), except that staining was visualized with Fast Red (Roche). For in situ hybridization on cleavage stage *Strigamia* embryos, the embryos were not dechorionated. After fixation, the stage 1 embryos were split into halves by inserting and opening a fine pair of tweezers through the centre of the egg. Later stage embryos were photographed in whole mount, stabilized in wells in 0.8% agarose, or flat mounted, in which case embryos were dissected away from the yolk with fine tweezers and flattened under a cover slip on a microscope slide. The ovary sac had to be dissected open before the hybridization, but otherwise an identical protocol was used to stain ovaries as for embryos in Chipman et al. (2004), except that staining was visualized with Fast Red (Roche). After in situ, ovaries were stained in 10 μg/ml DAPI solution in PBS overnight to reveal nuclei, and then transferred step-wise to 90% glycerol in PBS. Ovaries were mounted underneath a cover slip on a slide. Bright field images were taken on a Zeiss Axiophot compound microscope with a Leica DFC 300 FX camera. Fluorescent images and optical sections were taken using a Leica TCS SP5 confocal microscope. Brightness, contrast and colour balance of images were adjusted using Adobe Photoshop (version CS5).

### Antibody staining

All steps were at room temperature, unless otherwise noted. Ovaries were rehydrated from methanol into PBX (1× PBS and 0.5% Triton X-100) step-wise (25:50:75:100%). Ovaries were washed 4 times for 30 min each in PBX, then incubated in block solution (PBX, 5% normal goat serum and 1 mg/ml Bovine Serum Albumin) for 2 h, and incubated with primary antibody diluted in block overnight at 4 °C. We used rabbit anti-Vasa (gift of Akira Nakamura) at 1/2000 and mouse anti-NUP153 (Covance MMS-102P) at 1/100 dilution to stain the nuclear envelope. We washed 6 times for 20 min each in PBX and incubated again in block for 1 h, and then incubated with secondary antibody diluted in block overnight at 4 °C. We detected anti-Vasa with anti-rabbit-Alexafluor488 (Invitrogen/Molecular Probes) and anti-NUP153 with anti-mouse-Alexafluor568 (Invitrogen/Molecular Probes), both at 1/400 dilution. We washed for 4 times 20 min each in PBX, then stained with 10 μg/ml DAPI solution in PBX for 3 h to reveal nuclei, transferred to 50% glycerol in PBS and mounted under a cover slip. Images were taken as above.

## Results

### *Strigamia nanos* and *vasa* orthologues label primordial germ cells during embryogenesis

A set of 31 genes orthologous to genes known to have roles in germ line specification or differentiation in other species were identified in the *Strigamia* genome (Chipman et al., submitted). In order to find germ line markers, we screened 8 genes of this set for expression in the PGCs of *Strigamia* embryos (see the Methods section). We found that only the single *Strigamia vasa* orthologue and one of the two *nanos* orthologues, *nanos2*, showed unambiguous staining of cells that had been previously identified as germ cells at late stages of embryogenesis by Heymons (1901). Therefore *vasa* and *nanos2* were used for all further experiments. Note the *Strigamia* genome contains only a single copy of the *vasa* gene, but two copies of *nanos*, *nanos1* and *nanos2*. Both the *Strigamia nanos* paralogues possess the conserved double CCHC Zinc finger domain characteristic of this family, and show a two-to-one orthology to the single copy *nanos* genes present in most other arthropod genomes examined to date (Chipman et al., submitted).

**Fig. 1:**
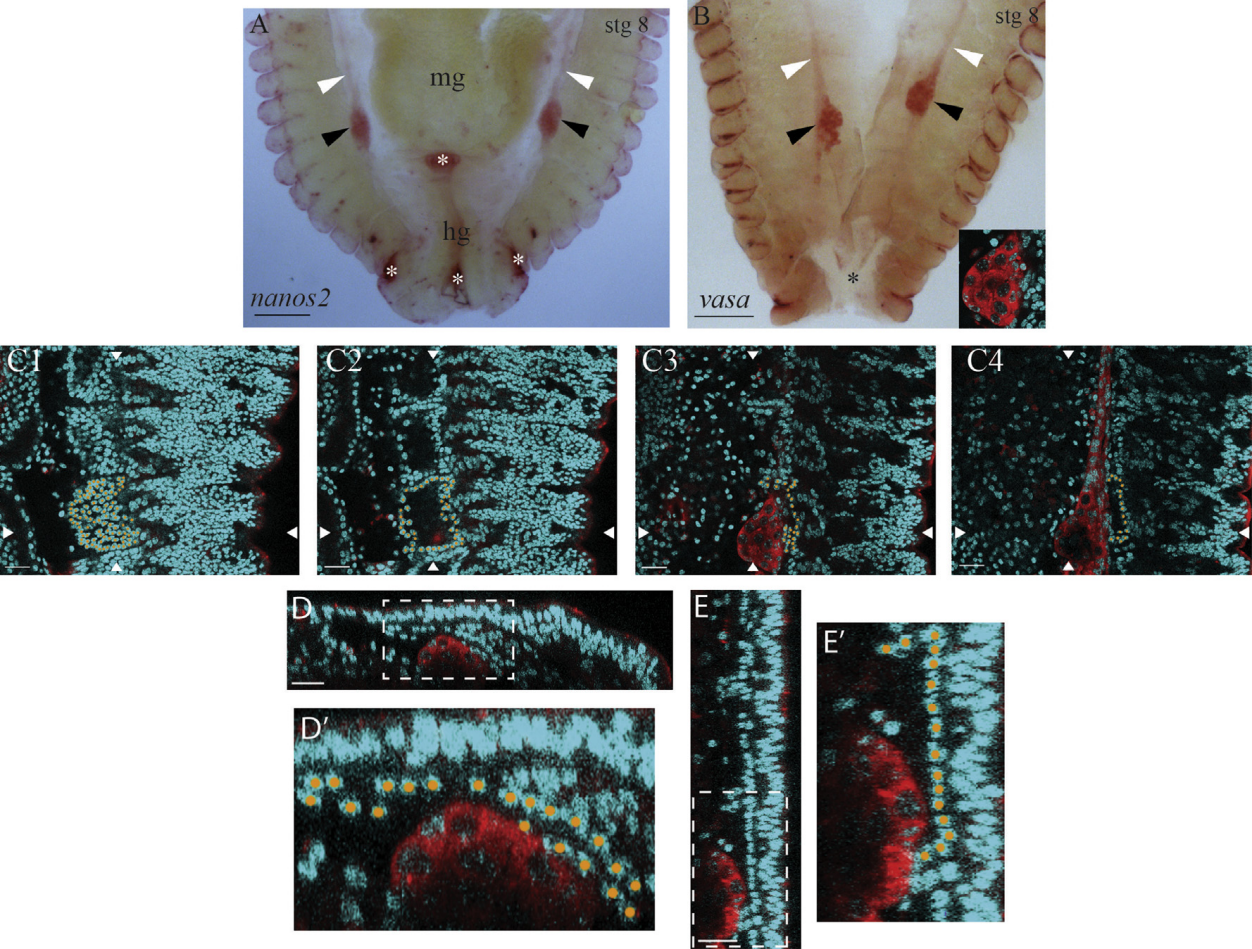
*vasa* and *nanos2* mark a pair of primordial germ cell clusters at the end of *Strigamia* embryonic development. (A) *nanos2* and (B) *vasa* transcripts (red) label a pair of primordial germ cell clusters, indicated by black arrowheads. A narrow strand of labelled cells extends from the anterior of this cluster, white arrowheads. Inset in (B) shows that the PGCs in the cluster are characterized by large, round nuclei with diffuse chromatin. Embryonic stage is indicated in the top right-hand corner. (C1)–(C4) A series of single optical sections moving from dorsal to ventral, at the position of the right-hand side *vasa*-positive cell cluster in the embryo in (B). The nuclei of the mesoderm in one of the segments are marked in orange. (C1) Dorsal-most view, shows the segmental organization of the putative gonadal mesoderm. (C2) Each segment of the gonadal mesoderm encloses a hollow cavity, which is derived from the prior coelomic pouches. (C3) The PGC cluster lies ventral to the mesoderm, and appears to be migrating into the embryonic gonad. (C4) Ventral-most view, shows the cluster and the narrow strand of labelled cells extending towards the anterior. (D) Transverse section at the horizontal level defined by the two arrowheads in (C). Superficial cells are oriented to the top. (E) Sagittal section at the vertical level defined by the two arrowheads in (C). Superficial cells are oriented to the right. (D′) and (E′) Enlarged views of the boxed areas in (D) and (E) respectively. As in (C), the mesodermal nuclei in one of the segments are marked in orange. A perforation is visible in the ventral wall of the mesodermal cavity, through which the PGC cluster is protruding. Anterior is to the top in all images. White asterisks in (A) indicate artefactual staining of the cuticle, and staining between segments in (B) is likely also artefactual. Black asterisk in (B) indicates an artefactual tear, where the hindgut and midgut tissue have been removed during dissection. Abbreviations: hg=hindgut; mg=midgut; stg=stage. Scale bar in (A, B) is 100 μm; in (C), (D) and (E) is 20 μm.

We find that transcripts of the *Strigamia nanos2* and *vasa* orthologues specifically label a pair of cell clusters on either side of the midgut at the end of embryogenesis (stage 8) (Fig. 1A and B). Using three lines of evidence, we argue that these cells are the *Strigamia* PGCs. First, they express conserved molecular markers of PGCs, *vasa* and *nanos2*. Second, they possess the morphological characteristics of PGCs, namely enlarged nuclei with diffuse chromatin (Fig. 1B, inset). Third, their final position at the end of embryogenesis is consistent with the expected association between the germ line and the embryonic gonad (Fig. 1C), described below. Their appearance and location is consistent with a previous description of centipede PGCs at this stage of embryonic development (Heymons, 1901). On this basis we consider these *nanos2*- and *vasa*-positive cells to be bona fide PGCs.

By examining a series of sections at the level of a *vasa*-positive cell cluster (Fig. 1C, D and E), it can be seen that the cluster is closely associated with a population of dorsal mesodermal cells. At the end of embryogenesis, Heymons describes the embryonic gonad as a number of segmentally organized chambers, each possessing an internal cavity derived from the prior coelomic cavities (Heymons, 1901). We observe that the dorsal mesodermal cells associated with the PGCs in *Strigamia* match this description, with a segmental organization of hollow chambers (Fig. 1C1 and C2; we have highlighted in orange the mesodermal nuclei of one of the segmental chambers). Therefore, by its appearance, position and association with PGCs, we identify this tissue as the mesoderm of the embryonic gonad.

We observe that the dorsal wall of each chamber is intact (Fig. 1C1 and D–E′), but the ventral wall is perforated (Fig. 1C2–C4 and D–E′). The PGCs appear to be migrating through the perforations in the ventral wall into the cavity (Fig. 1C3 and D–E′). It is noticeable that the PGCs have a distinctive tissue-level organization; the cells are aggregated into a globular cluster at the posterior end (Fig. 1A and B, black arrowheads; high magnification view in C3) and a narrow strand of cells extends from the anterior of this cluster (Fig. 1A and B, white arrowheads; high magnification view in C4). This will be described further below.

### PGCs are specified early in embryonic development and accumulate at the blastopore

**Fig. 2:**
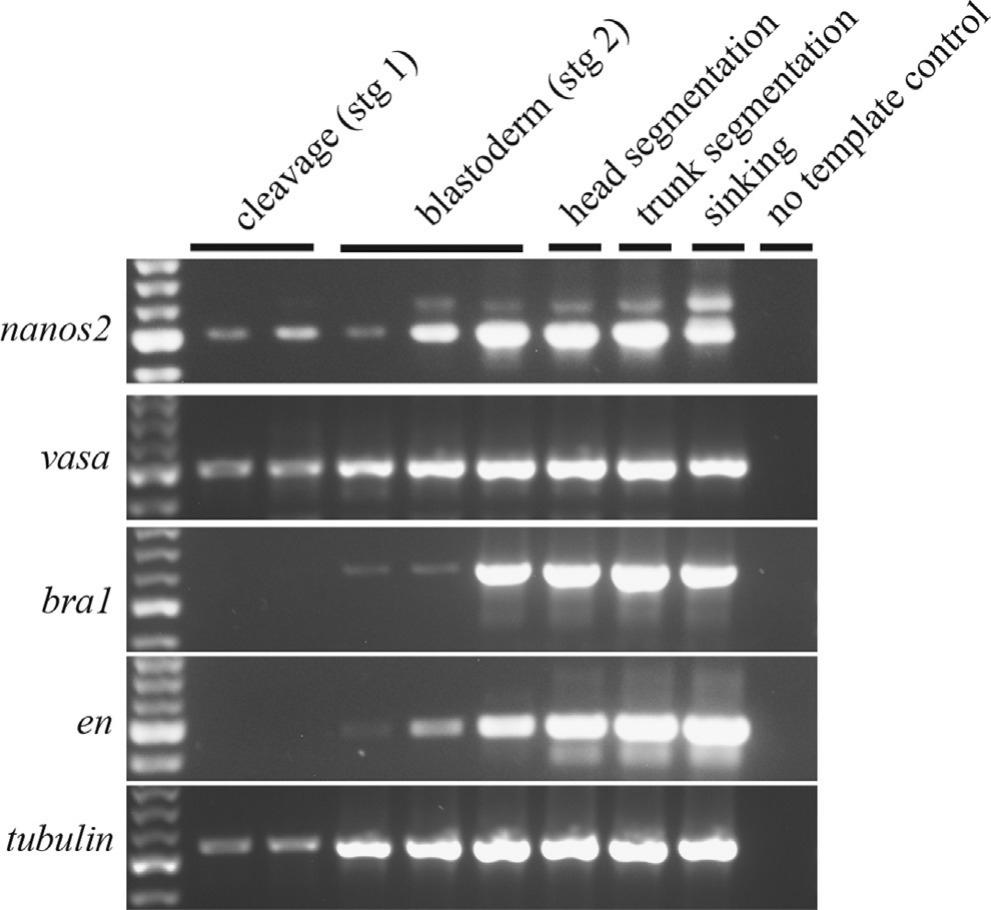
*vasa* and *nanos2* are expressed from cleavage stages onwards. RT-PCR results. 5 different embryonic stages of *Strigamia* were examined for the presence or absence of transcripts of 5 genes: *nanos2*, *vasa*, *brachyury1* (*bra1*), *engrailed* (*bra1* and *en* selected as negative controls for stage 1) and *alpha tubulin* (positive control). *nanos2* and *vasa* transcripts are detected in cleavage stage embryos, but *bra1* and *en* transcripts are not detected until the blastoderm stage. Head segmentation=stage 3 embryos; trunk segmentation=stage 4 embryos; sinking=stage 6 embryos. Each sample is pooled RNA from a single clutch of eggs. Note that a constant proportion of RNA from each clutch, regardless of stage, was used in the RT-PCR. Therefore at least part of the increase in band intensity for later stage clutches is due to a larger total input of RNA in the RT-PCR.

To document the first appearance and subsequent development of the PGCs, we first determined the earliest embryonic stage at which *vasa* and *nanos2* transcripts could be detected. Using RT-PCR, we detect both *vasa* and *nanos2* transcripts in cleavage stage (stage 1) *Strigamia* embryos, before blastoderm formation (Fig. 2). We infer that these are maternal or very early zygotic transcripts. In contrast, transcripts of *Strigamia brachyury* and *engrailed* orthologues (*bra1* and *en*) are first detected at blastoderm stage (stage 2). These genes were selected as controls, based on in situ hybridization data that showed they are first expressed after blastoderm formation.

**Fig. 3:**
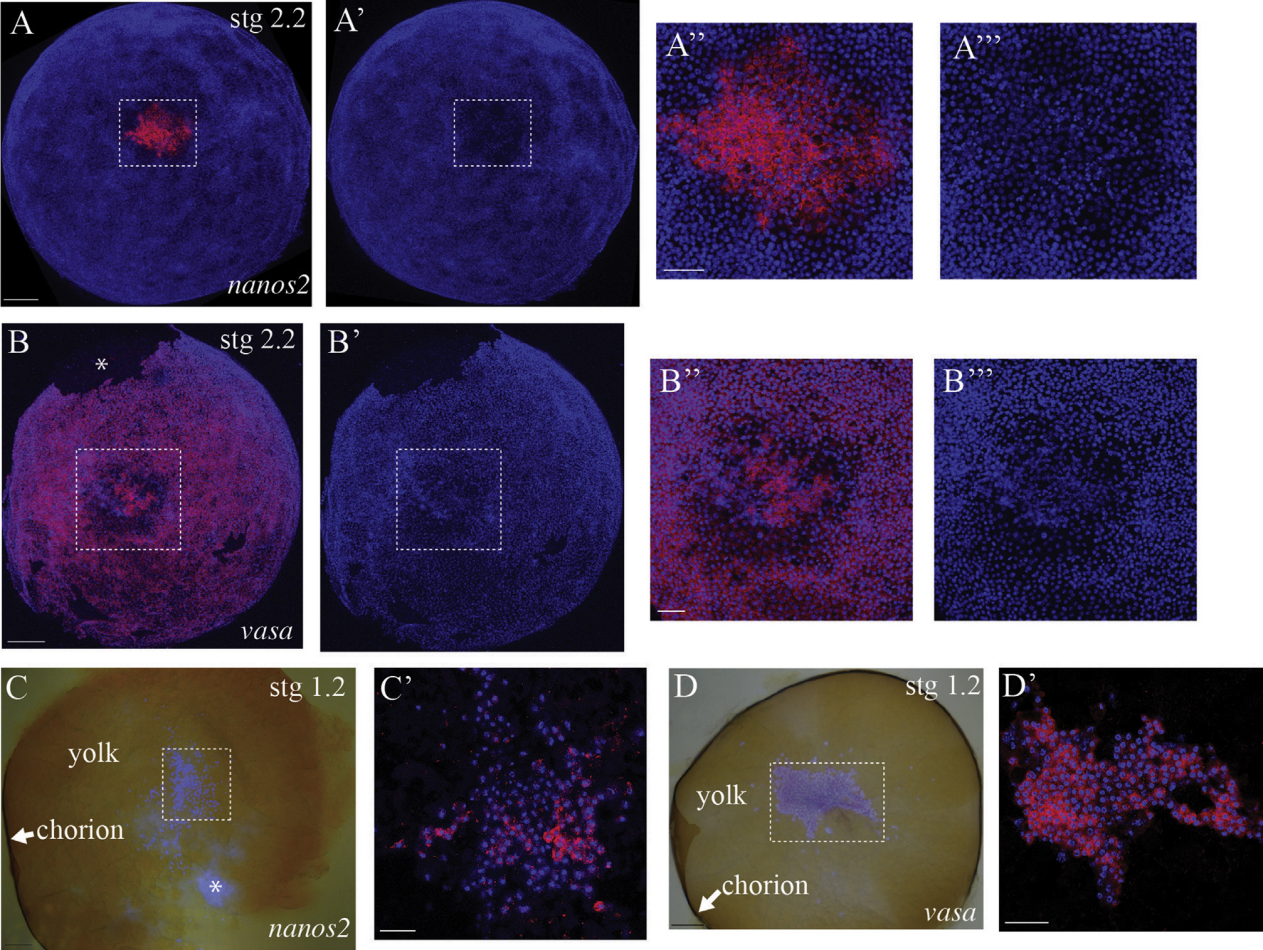
PGCs are specified early in embryonic development and accumulate at the blastopore. (A) and (B) Blastoderm stage embryos (stage 2.2) in which expression (red) is overlain on a nuclear stain (blue) to show the overall morphology of the embryo. Posterior pole/blastopore view. (A) *nanos2* is expressed exclusively in cells lying within the blastopore. (B) *vasa* is expressed almost ubiquitously across the blastoderm, but is expressed differentially in cells lying within the blastopore (see text). (A′), (B′), (A‴) and (B‴) show the nuclear stain without the expression to emphasize the distinctive low cell density that characterizes the blastopore. (A″), (A‴), (B″) and (B‴) Enlarged views of the boxed area in (A), (A′), (B) and (B′) respectively. (C) and (D) Cut surface of a bisected cleavage stage egg, still held within its half of the chorion to maintain integrity. At this stage, all cells lie deep within the yolk, where they undergo multiple rounds of cell division. Images are an overlay of a bright field and a fluorescent image to highlight the position of the cells (visible as DAPI-stained nuclei) deep inside the yolk. The two embryos in (C) and (D) are different. (C′) Confocal section taken at the approximate position of the boxed area in (C). *nanos2* transcripts (red) are localized to a subpopulation of cleavage cells. (D′) Confocal section taken at the approximate position of the boxed area in (D). *vasa* transcripts (red) are detected in many, if not all, cleavage cells at variable levels. There is no clear, overall pattern of spatial localization. Embryonic stage is indicated in the top right-hand corner. White asterisk in (B) indicates an artefactual tear; white asterisk in (C) indicates an optical artefact due to the yolk. Abbreviations: stg=stage. Scale bar in (A), (B), (C) and (D) is 100 μm; in (A″), (B″), (C′) and (D′) is 50 μm.

With this result, we next examined the expression of *vasa* and *nanos2* during blastoderm stage embryos. The newly formed blastoderm is an approximately uniform monolayer of cells (stage 2.1; Brena and Akam, 2012). Later one hemisphere of the blastoderm becomes multi-layered (stage 2.2). This multi-layered region is centred on a circular area that remains covered by only a single layer of cells. The lower density of nuclei in this circular area distinguishes it from the surrounding tissue. This area has been described previously as the blastopore (Brena and Akam, 2012). The earliest, unambiguous accumulation of PGCs we have observed is at the blastoderm stage in cells lying within the blastopore. This is shown by the localization of *nanos2* transcripts, which are expressed exclusively in cells lying within the blastopore (Fig. 3A–A‴). *vasa* transcripts, on the other hand, are expressed almost ubiquitously across the blastoderm, but are expressed differentially in cells lying within the blastopore (Fig. 3B–B‴). One subset of blastoporal cells that tend to lie around the outer margin of the blastopore express negligible or low levels of *vasa* transcript, while a second subset that lie approximately in the centre of the blastopore express higher levels of transcript, comparable to that visible in the rest of the blastoderm (Fig. 3B–B‴).

Given the association between the PGCs and the blastopore at the blastoderm stage, we wanted to determine if *vasa* or *nanos2* transcripts showed any localized expression before this, during cleavage (stage 1). In *Strigamia*, a large number of cleavage cells are generated as a mass near the centre of the egg. These subsequently migrate between the yolk pyramids to the surface (Brena and Akam, 2012).

We performed in situ hybridization on the split halves of cleavage stage embryos, before cells reach the surface of the egg. In all embryos examined, the majority of cleavage cells do not express *nanos2*, but in most embryos (*N*=11/16) we observe a subpopulation of *nanos2*-positive cells (Fig. 3C and C′). These *nanos2*-positive cells sometimes appear as a single cluster, sometimes in small patches or as single cells dispersed among the *nanos2*-negative cells. *vasa*, on the other hand, is expressed in most, if not all, cleavage cells in all embryos examined (*N*=10/10) (Fig. 3D and D′). The level of *vasa* expression varies between cells, and shows no clear pattern of spatial localization (Fig. 3D and D′). Unfortunately the developmental stages where the cleavage cells migrate to the surface of the egg and spread across the yolk to form a blastoderm (stages 1.3–1.4) are extremely fragile and difficult to handle, and it has not been possible to determine the relationship between the *nanos2*-positive cell population during cleavage and that at the blastopore.

### The blastopore is a site of dynamic *brachyury* expression

In order to understand the relationship between the PGCs and the blastopore, we needed a more complete understanding of the development and ultimate fate of the blastopore. To achieve this we investigated the expression of a *Strigamia brachyury* orthologue, *bra1*, one of two *brachyury* orthologues present in the *Strigamia* genome (Chipman et al., submitted).

**Supplementary Fig. S2:**
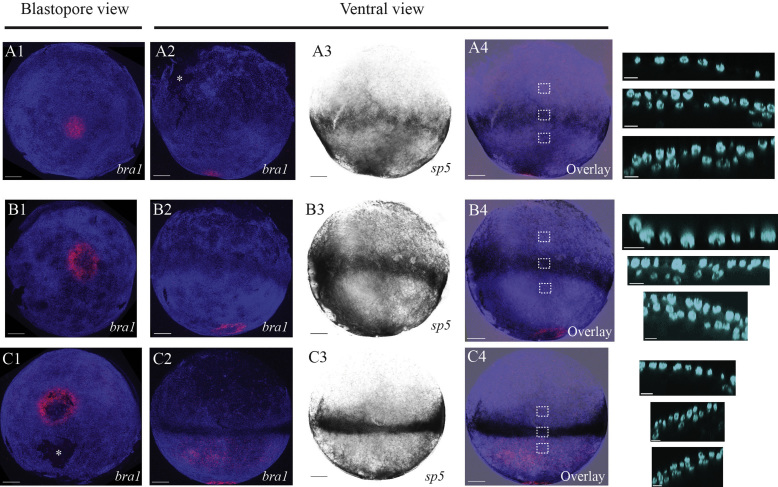
**Deep*****brachyury1*****-positive cells in the posterior blastoderm and early germ band.** (A) and (B) Fluorescent overlay of *bra1* expression (red) and nuclear stain (blue). Posterior pole/blastopore views. Embryonic stage is indicated at the top right-hand corner. The arc of *bra1* expression around the anterior margin of the blastopore is visible, as seen in embryos at approximately the same stage in Fig. 4D and E. By increasing the brightness, weak, diffuse but specific staining is visible throughout the posterior part of the multi-layered blastoderm (A) or forming germ band (B). Note this staining is restricted to the embryonic territory, and is absent from the extra-embryonic cells. (A′) and (B′) Panels show high magnification transverse sections in the approximate regions indicated by the boxed areas in (A) and (B) respectively. Sections show that the diffuse *bra1* staining is restricted to deep cells, which are putative mesodermal cells. We hypothesize that at least some of the *bra1*-expressing cells in the arc around the blastopore internalize at the blastopore and migrate anteriorly to form mesoderm, but that they down-regulate, and ultimately switch off, *bra1* expression as they do so. In (A) and (B) scale bar is 100 μm; in (A′) and (B′) is 10 μm.

**Fig. 4:**
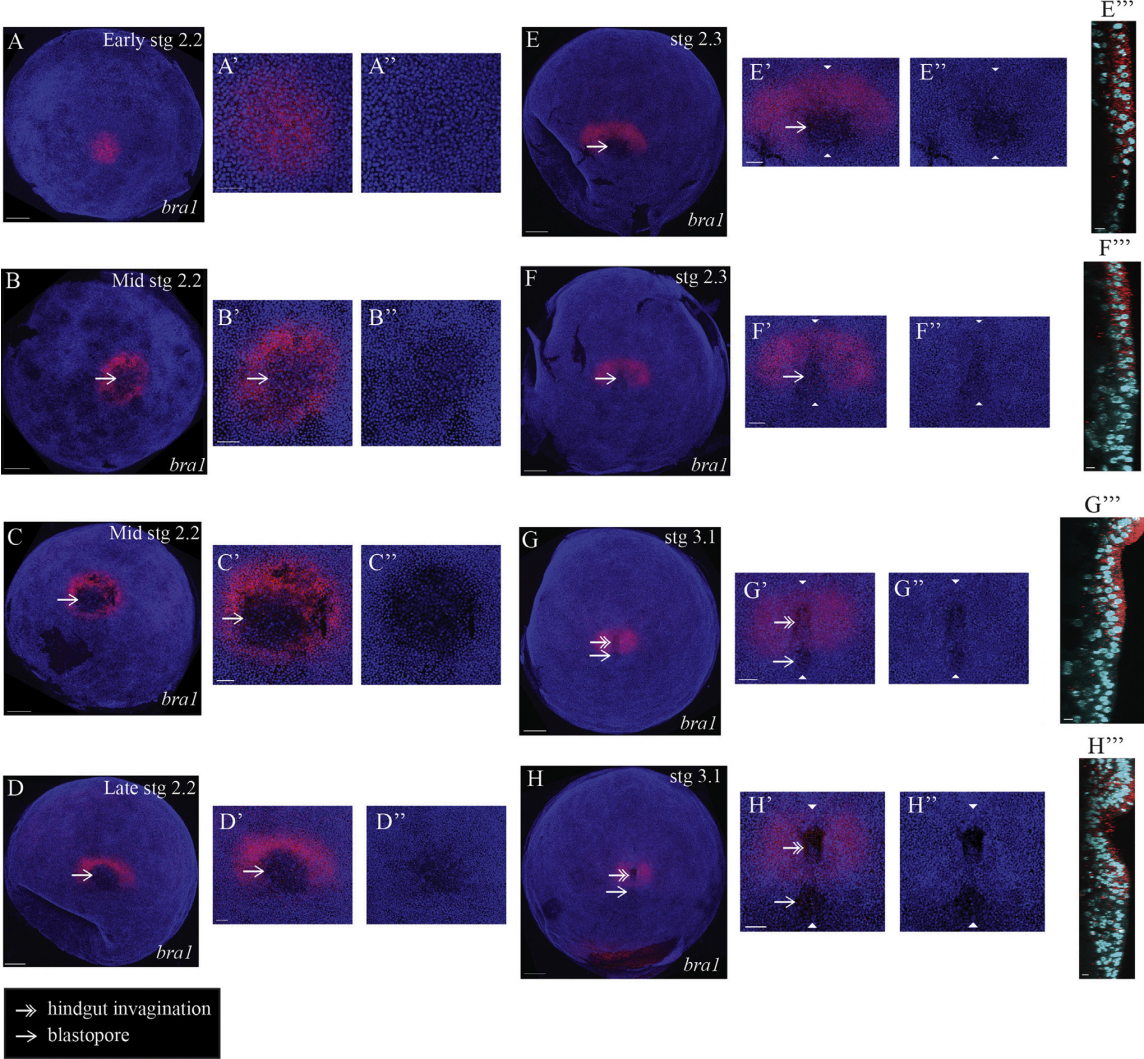
The blastopore is a site of dynamic *brachyury* expression. (A) *bra1* expression is first detected as a circular patch that precedes the distinctive morphology of the blastopore. (B) *bra1* expression resolves into a ring that surrounds the blastopore as the distinctive low cell density begins to appear. Blastopore is indicated with a single-headed arrow throughout the rest of the figure. (C) The diameter of the blastopore reaches its maximum extent and the density of cells within it is at a minimum. (D) *bra1* further resolves into an arc at the anterior margin of the blastopore. (E) The two halves of the semi-circular patch of *bra1* expression move closer together. (E‴) The hindgut invagination has not yet commenced in the *bra1*-expressing territory and the blastopore remains covered by only a single layer of cells. (F) The outline of the blastopore transforms from a circular to a triangular appearance. (F‴) *bra1*-negative deep cells are now visible underneath the blastopore. (G) The hindgut invagination has begun, and is marked with a double-headed arrow for the rest of the figure. (G‴) A shallow pit is visible within the *bra1*-positive territory that is the nascent hindgut cavity. (H) and (H‴) The hindgut invagination continues to deepen. A mixture of superficial and deep cells remains visible at the blastopore. (A)–(H) Fluorescent images are an overlay of *bra1* expression (red) and nuclear stain (blue) to show the morphology of the embryo. Embryonic stage is indicated in the top right-hand corner. All images are posterior pole/blastopore views. Anterior is to the top in (D)–(H). For embryo in (D), before the appearance of the head condensation, we define the anterior–posterior axis by the relative position of the future hindgut invagination (anterior) and the blastopore (posterior). This is consistent with the anterior–posterior axis defined by the location of the head condensation, which becomes clear in stage 2.3. Each main image is accompanied by two panels (A′)–(H′) and (A″)–(H″) that are enlarged views of the corresponding blastopore area in (A)–(H) respectively. (A″)–(H″) Panels show the nuclear stain without the expression to reveal the changes in cell density and blastopore diameter that occur over this developmental period. (E)–(H) are accompanied by a third panel (E‴)–(H‴) that shows a sagittal section at the vertical level defined by the two white arrowheads in (E′)–(H′) and (E″)–(H″). Superficial cells are oriented to the right. Abbreviations: stg=stage. Scale bar in (A)–(H) is 100 μm; in (A′)–(H′) is 50 μm; in (E‴)–(H‴) is 10 μm.

The earliest *bra1* expression we detect is a circular patch in a blastoderm stage embryo (Fig. 4A; onset of stage 2.2; embryos in Fig. 4A–C staged using an independent molecular marker, see Fig. S1). This expression precedes the distinctive low cell density that characterizes the future blastopore region (Fig. 4A′ and A″). At least part of the blastoderm is multi-layered by the time of appearance of this circular patch (Fig. S1). Later, *bra1* transcripts clear from the centre of this patch outwards, and thus resolve into a ring of expression surrounding the blastopore (Fig. 4B and C; mid stage 2.2; the blastopore is now characterized by a lower cell density and is indicated with a single-headed arrow throughout the rest of the figure). The appearance of the low cell density is gradual and coincides with this transition from a patch to a ring of expression (Fig. 4B′, B″, C′ and C″). The diameter of the blastopore reaches its maximum extent at this stage and the cell density within the blastopore is at its lowest (Fig. 4C–C″). It appears that at least some of the *bra1*-expressing cells internalize to form mesoderm, but that they down-regulate, and ultimately switch off, *bra1* expression as they do so (Fig. S2).

In late stage 2.2 embryos, the posterior part of the *bra1* ring fades, and thus resolves into an arc of expression around the anterior margin of the blastopore (Fig. 4D). At the end of stage 2 (stage 2.3), the future head condenses at the anterior, ventral side of the blastoderm, and a recognizable germ band begins to form (Brena and Akam, 2012). During this stage, the *bra1* expression remains as a semi-circular patch, but the diameter of the semi-circle narrows, and the left and right halves of the expression move closer together (Fig. 4E and F). At the same time, the blastopore transforms from a circular to a triangular outline (Fig. 4F′ and F″).

As segmentation begins in the head at the onset of stage 3, the diameter of the blastopore continues to narrow and the hindgut begins to invaginate within the *bra1*-expressing territory (Fig. 4G and H; the hindgut invagination is indicated with a double-headed arrow). From this time onwards the hindgut epithelium is marked by continuous *bra1* expression (data not shown), much as it is in insects (Berns et al., 2008; Kispert et al., 1994; Shinmyo et al., 2006).

### PGCs internalize through the closing blastopore, and not through the hindgut

**Fig. 5:**
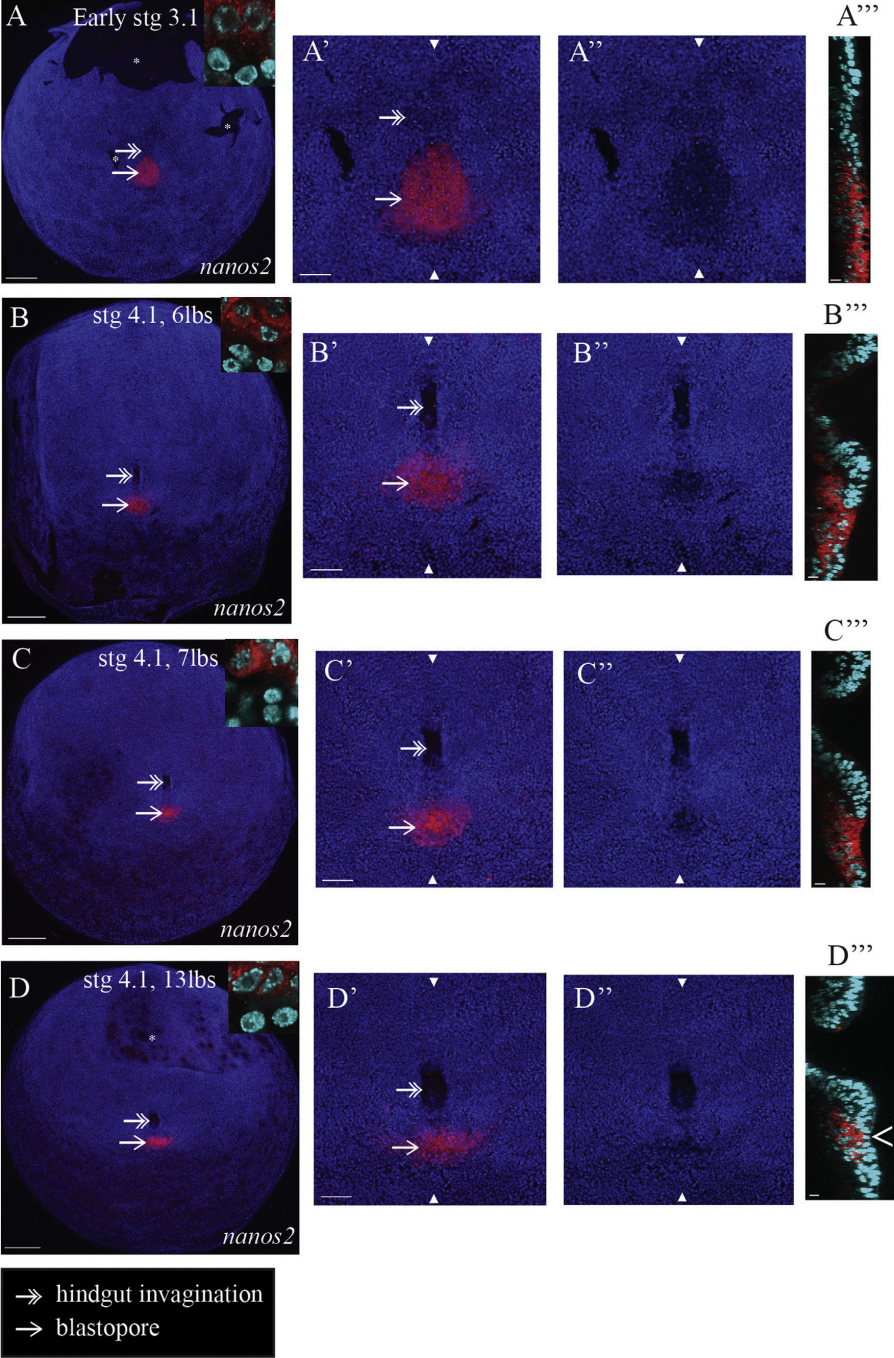
PGCs internalize through the closing blastopore. (A) *nanos2*-expressing cells lie within the blastopore. The hindgut invagination has just begun, forming a very shallow depression. (B) The hindgut invagination is clearly visible and has already formed a relatively deep pocket. The population of *nanos2*-poisitive cells is clearly posterior to and outside of the hindgut, in the remaining less distinct area of low cell density. Within the narrowing area of the blastopore, some *nanos2*-positive cells are located underneath non-labelled cells, while others remain at the surface. (C) The nuclei of these superficial cells appear to be displaced basally. (D) Finally the entire cluster of *nanos2*-positive cells lies underneath a layer of non-labelled cells (hollow arrowhead), such that they are now internal with respect to the ectoderm. It appears that the blastopore has closed over the *nanos2*-positive cells. Also, the initially circular patch of *nanos2*-expressing cells has transformed into a transverse arc of cells. (A)–(D) Images are an overlay of *nanos2* expression (red) and nuclear stain (blue) to show the morphology of the embryo. Posterior pole/blastopore views, with anterior to the top. Embryonic stage is indicated at the top of each main image. Insets are high magnification views of the *nanos2*-positive cells in comparison with neighbouring mesodermal cells. Three panels accompany each main image to the right. (A′)–(D′) and (A″)–(D″) Panels are enlarged views of the corresponding blastopore area in (A)–(D). (A″)–(D″) Panels show the nuclear stain without *nanos2* expression to emphasize morphological changes. (A‴)–(D‴) Panels show a sagittal section at the vertical level defined by the two white arrowheads in (A′)–(D′) and (A″)–(D″). Superficial cells are oriented to the right. Note that the progression of PGC internalization is somewhat variable with respect to segment addition, implying that the two processes are not tightly correlated between embryos. Progression of hindgut invagination is a more reliable staging marker for this process. Abbreviations: lbs=leg-bearing segments; stg=stage. Scale bar in (A), (B), (C) and (D) is 100 μm; in (A′), (B′), (C′) and (D′) is 50 μm; in (A‴)–(D‴) is 10 μm.

We examined the further development of the PGCs in relation to the transformation of the blastopore and the invagination of the hindgut. In contrast to the behaviour of the PGCs in *Drosophila melanogaster* (Campos-Ortega and Hartenstein, 1985; Sonnenblick, 1941), we observe that the hindgut invagination does not carry the *Strigamia* PGCs into the body cavity (Fig. 5).

The PGCs continue to lie within the blastopore during stage 3.1 (Fig. 5A–A‴). When the process of PGC internalization starts during early trunk segmentation (early stage 4.1; Fig. 5B), the hindgut invagination, which began to form earlier (stage 3.1; Fig. 5A and Fig. 4G and H), has already formed a relatively deep pocket (Fig. 5B‴). Significantly, the population of *nanos2*-positive cells is clearly posterior to and outside of the hindgut, in the remaining (and now less distinct) area of low cell density (Fig. 5B–B‴). We never observe *nanos2*-positive cells inside the hindgut cavity. Within the narrowing blastopore area, some *nanos2*-positive cells remain at the surface, while others are observed underneath non-labelled cells (Fig. 5B‴ and C‴). At around the end of stage 4.1, the blastopore closes over the germ cells. The entire cluster of *nanos2*-expressing cells now lies completely inside the embryo (Fig. 5D). A layer of *nanos2*-negative cells overlies and seals off the *nanos2*-positive cells beneath (Fig. 5D‴, hollow arrowhead).

Therefore, by following *nanos2* expression across a closely staged series of embryos, we find that the PGCs become internalized as the blastopore closes, and that this occurs at a distinct location and time from the invagination of the hindgut.

The characteristic nuclear morphology (enlarged nuclei with more diffuse chromatin) observed in the later PGCs begins to appear over this developmental period (stage 3 to 4.1; Fig. 5, insets). However, the differences between the PGCs and surrounding cells are not pronounced at this stage, and are variable between PGCs.

### PGCs migrate to the embryonic gonad

**Fig. 6:**
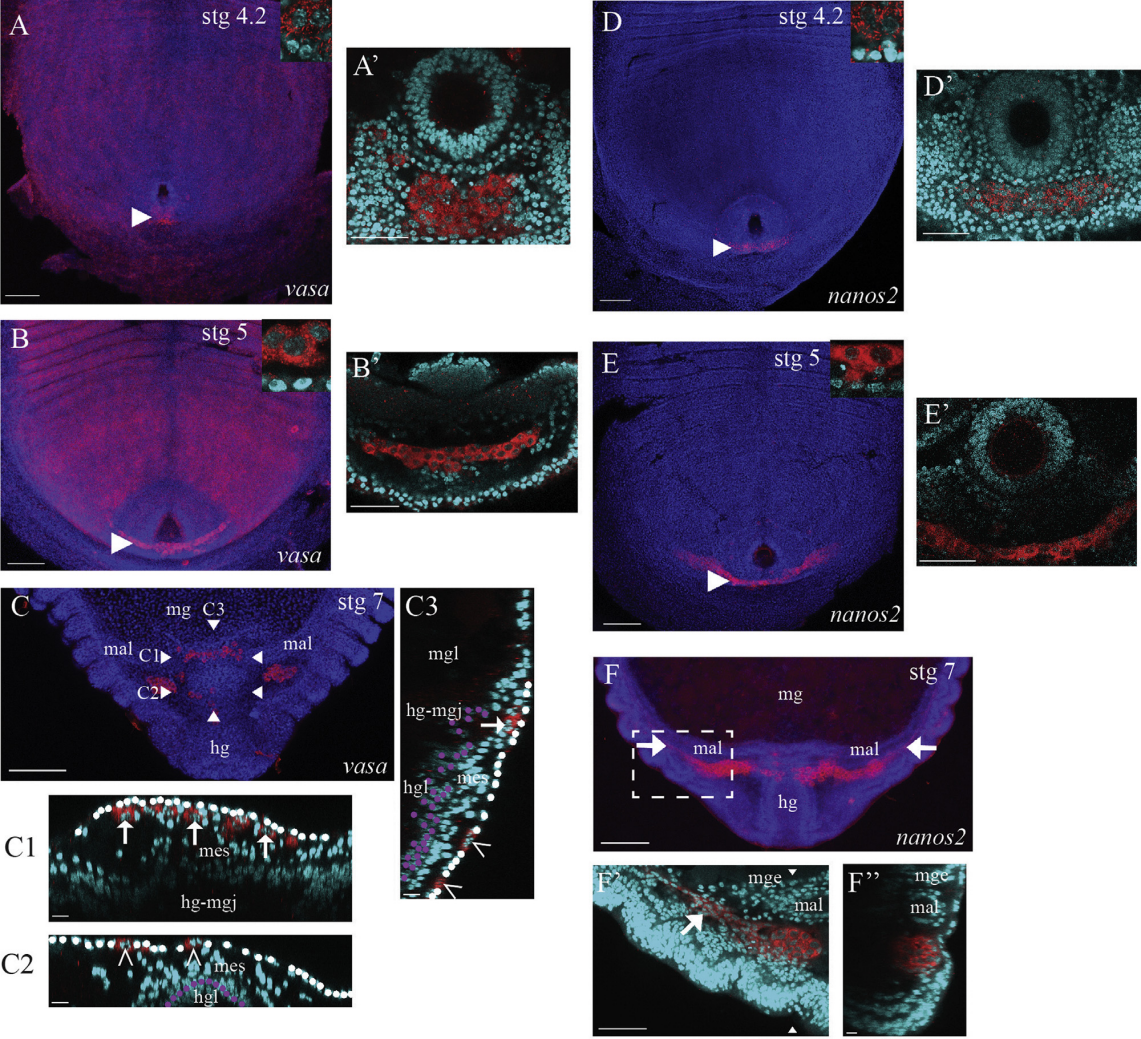
PGCs migrate to the embryonic gonad. (A) and (B) *vasa*- or (D) and (E) *nanos2*-positive primordial germ cells (arrowheads) reside at the posterior tip of the germ band during stages 4 and 5, spanning the midline. Note the broad expression of *vasa* transcripts in somatic cells throughout the embryo visible in (A) and (B). Insets show the marked differences in nuclear morphology between the labelled germ cells and non-labelled surrounding cells, which are very pronounced by stage 5. (A′), (B′), (D′) and (E′) High magnification views of the PGCs indicated with arrowheads in (A), (B), (D) and (E). The PGCs lie posterior to the hindgut, underneath the ectoderm and surrounded by mesodermal cells. (C) PGCs have begun to migrate. In the overview image, PGCs are visible at three sites: at the hindgut–midgut junction, overlying the hindgut, and as bilateral clusters on either side of the dorsal midline. (C1)–(C3) Nuclei of the dorsal extra-embryonic epithelium are marked in white and nuclei of the hindgut epithelium are marked in purple. (C1) Transverse section at level of the PGCs at the hindgut–midgut junction; defined by the horizontal line, marked (C1), connecting the two arrowheads in (C). PGCs (arrows) are located out of the plane of the overlying dorsal epithelium (white) and are intermingled with mesodermal cells. (C2) Transverse section at level of the PGCs overlying the hindgut; defined by the horizontal line, marked (C2), connecting the two arrowheads in (C). PGCs (hollow arrowheads) are located in the plane of the dorsal epithelium (white). (C3) Sagittal section along the hindgut; defined by the vertical line, marked (C3), connecting the two arrowheads in (C). More posterior PGCs (hollow arrowheads) are found within the dorsal epithelium (white), but more anterior PGCs (arrows) appear to have detached and entered the mesoderm. (F) The PGCs have split into bilateral clusters on either side of the dorsal midline, and some cells have begun to migrate more anteriorly through the mesoderm. The PGCs adopt a ‘clump and strand’ tissue-level morphology; strand is indicated in (F) and (F′) with a white arrow. (F′) Enlarged view of the boxed area in (F), showing the differences in nuclear morphology between the clump cells, the strand cells and the surrounding mesodermal cells. (F″) Sagittal section at the vertical line defined by the two arrowheads in (F′). The globular nature of the clump cells can be seen. (A)–(F) Fluorescence images with expression (red) and nuclear stain (blue) overlain. Posterior trunk views, with anterior to the top. Embryonic stage is indicated at the top of each main image. In transverse sections, superficial cells are oriented to the top; in sagittal sections, to the right. Abbreviations: mes=mesoderm; mg=midgut; mge=midgut epithelium; mgl=midgut lumen; mal=Malpighian tubule; hg=hindgut; hgl=hindgut lumen; hg-mgj=hindgut–midgut junction; stg=stage. Scale bar in (A), (B), (C), (D), (E) and (F) is 100 μm; in (A′), (B′), (D′), (E′) and (F′) is 50 μm; in (C1)–(C3) and (F″) is 10 μm.

During stages 4 and 5 of *Strigamia* embryonic development, the PGCs remain at the posterior tip of the germ band as a single cluster lying just beneath the ectoderm, spanning the midline (Fig. 6A, B, D and E). The differences in nuclear morphology from surrounding mesodermal cells become increasingly pronounced over stage 4, and by stage 5 the PGCs are very distinct (Fig. 6A, B, D and E, insets).

During stages 6 and 7, the PGCs begin to migrate anteriorly, in the direction of the hindgut–midgut junction, and laterally to form bilateral clusters on either side of the dorsal midline (Fig. 6C and F).

In some embryos we observe that the PGCs appear to disperse from the posterior cluster and migrate as individual cells (Fig. 6C). At stage 7 definitive dorsal closure is not complete, but an extra-embryonic epithelium overlies the mesoderm and the hindgut tube dorsally (Fig. 6C1, C2 and C3; nuclei of the dorsal extra-embryonic epithelium are highlighted in white, and nuclei of the hindgut epithelium are highlighted in purple). The cells appear to migrate through this dorsal epithelium towards the anterior. In the posterior, the cells are found in the plane of the epithelium (Fig. 6C2 and C3, hollow arrowheads), but once they reach the vicinity of the hindgut–midgut junction, they appear to detach from the epithelium and move into the mesoderm (Fig. 6C1 and C3, arrows).

We also observe that the PGCs split and sort into bilateral clusters (Fig. 6F; this process is also visible in 6C). Each cluster resides in a mesoderm-filled cavity bounded by the hindgut, the midgut epithelium and the ectoderm. The Malpighian tubules are also developing at this time, and are visible running through this cavity alongside the midgut epithelium (Fig. 6F–F″: mal). The PGCs adopt a distinctive tissue-level organization: each cluster has a compact clump of cells at its posterior, with a long, narrow strand of cells extending anteriorly from this (Fig. 6F and F′, strand indicated with white arrows). The cells in the clump possess the distinctive enlarged nuclei, but the strand cells lack this feature and their nuclear morphology appears indistinguishable from neighbouring mesodermal cells (Fig. 6F′).

By stage 8 the PGCs arrive at the putative embryonic gonad, as described above. The ‘clump and strand’ tissue morphology is still clearly visible (Fig. 1A and B). At stage 7, the position of the PGCs is approximately 1–2 segment widths anterior to the posterior tip of the germ band (Fig. 6C and F). By stage 8, the PGCs lie around 6–8 segment widths anterior to the posterior tip (Fig. 1A and B). The PGCs and gonadal mesoderm undergo further post-embryonic development, but description of these processes is beyond the scope of this paper.

### No clear evidence for a maternal germ plasm in developing oocytes

The localized expression of *nanos2* in only some of the cleavage cells (Fig. 3C) suggests the possibility that germ cells may be specified by a localized germ plasm present in the egg. We cannot perform in situ hybridization on mature yolky eggs, but we have tested whether *vasa* or *nanos2* transcripts are asymmetrically localized in developing oocytes.

**Fig. 7:**
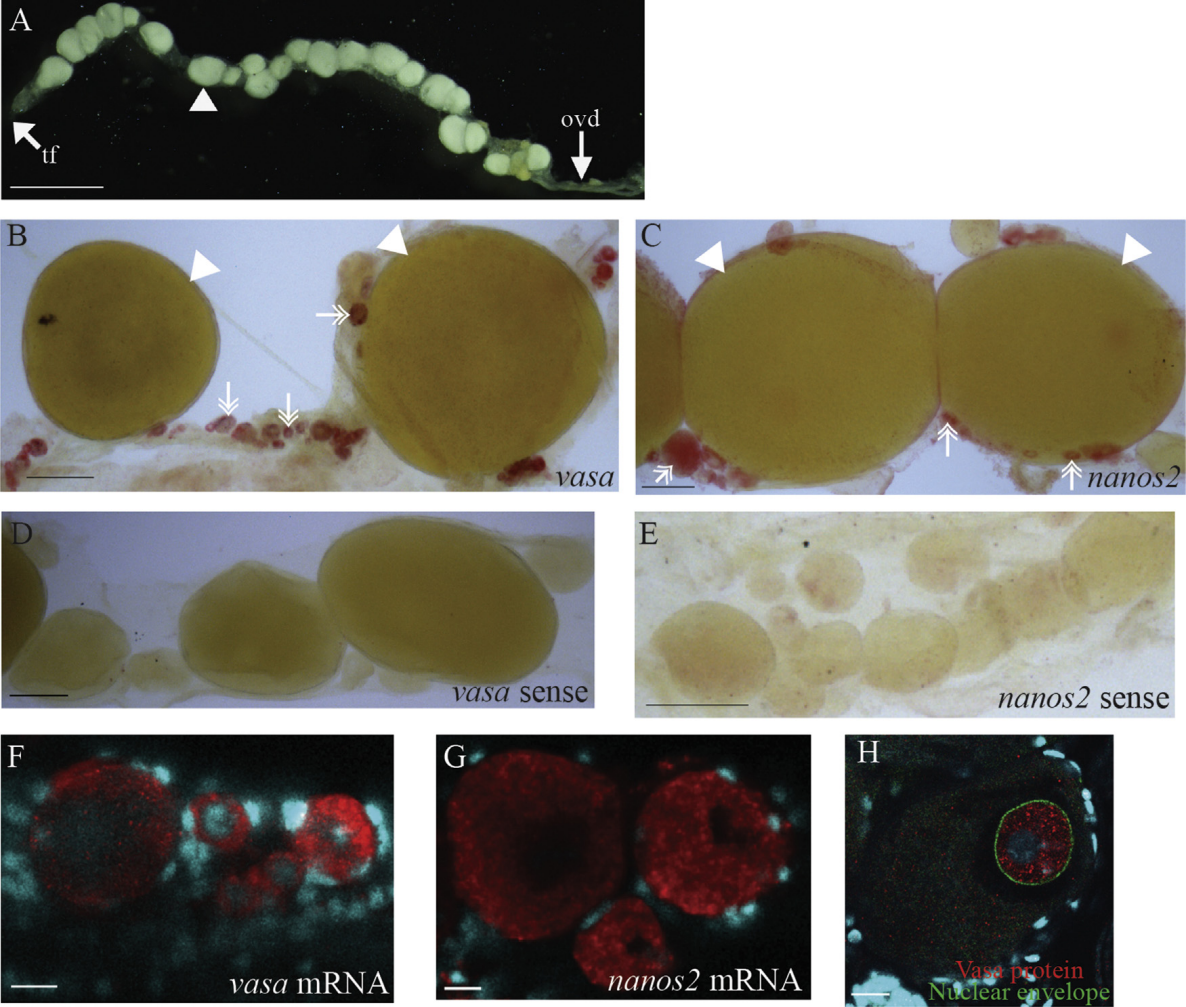
*vasa* and *nanos2* expression in developing oocytes. (A) Overview image of the single, unpaired ovary of an adult *Strigamia maritima* female (dissected from an individual collected in October 2013; ovaries collected in June 2013 had fewer large oocytes). Anterior is to the left. Only the large, yolky oocytes are clearly visible in this image; one of which is labelled with an arrowhead. (B) *vasa* or (C) *nanos2* transcripts (red) are expressed in oocytes at a range of different stages; examples of which are indicated with double-headed arrows. Large, yolky oocytes (indicated with arrowheads) are not stained (see text). (D) *vasa* or (E) *nanos2* sense probe gives no staining. (F) *vasa* or (G) *nanos2* transcripts (red) show a uniform distribution in the oocyte cytoplasm. Follicle cells are not stained. (H) Ovary is double-labelled for Vasa protein (red) and the nuclear envelope (green). Vasa is localized inside the germinal vesicle of the oocyte. It is excluded from a circular region within the nucleus that shows faint DAPI staining, which we infer to be the nucleolus. Vasa is either absent or expressed at low, uniform levels in the oocyte cytoplasm. (F)–(H) Ovaries counter-stained with DAPI to reveal nuclei (cyan). Abbreviations: ovd=oviduct; tf=terminal filament. Scale bar in (A) is 1 mm; in (B)–(E) is 100 μm; in (F)–(H) is 10 μm.

Adult centipedes possess a single, medial ovary that lies dorsal to the midgut (Lewis, 1981; Minelli, 2011) (Fig. 7A). In the adult ovaries examined, *vasa* and *nanos2* transcripts, and Vasa protein, were detected in developing oocytes at a range of different stages (Fig. 7B, C and F–H). A control in situ hybridization with sense probes shows no staining (Fig. 7D and E).

Oocytes of very different stages are found together at all positions in the ovary—the progression of oogenesis shows no clear anterior–posterior organization (Fig. 7A–C; large, yolky oocytes are indicated with arrowheads). Using in situ hybridization or antibody stains, we find that all oocytes below 15 μm, and no oocyte above 130 μm in diameter, showed staining. The staining of oocytes at sizes in between these two thresholds was variable (Fig. 7B and C; examples of stained oocytes at different sizes are indicated with double-headed arrows). In previous studies of centipede oogenesis, a membrane of glycoproteins has been described that forms around the developing oocyte, before the deposition of the mature chorion (Herbaut, 1974; Lewis, 1981; Miyachi and Yahata, 2012). We consider it likely that the formation of such a membrane occurs during *Strigamia* oogenesis, and that this impedes penetration of the in situ probe or antibody into later oocytes. We infer that the timing of the formation of this membrane is somewhat variable with respect to oocyte size. In addition, the decline in detectable signal from larger oocytes is probably compounded by the dilution of cytoplasm through the accumulation of yolk.

Nonetheless, in all successfully stained oocytes, we observe a uniform distribution of *vasa* and *nanos2* transcripts in the oocyte cytoplasm (Fig. 7F and G). Vasa protein is primarily localized within the germinal vesicle of the oocytes, but excluded from the nucleolus (Fig. 7H). It is either absent, or only detectable at low, uniform levels in the oocyte cytoplasm (Fig. 7H). On the basis of these data, we find no evidence for a specialized region of the oocyte cytoplasm that might correspond to a germ plasm. However, we cannot rule out the possibility that other putative germ plasm transcripts or proteins that we have not analysed here might be localized in one region of the oocyte cytoplasm.

## Discussion

### Centipede primordial germ cells are specified much earlier in development than previously reported

By examining the expression of *vasa* and *nanos* orthologues in the centipede *S. maritima*, we demonstrate that the primordial germ cells are specified from at least the blastoderm stage, and possibly earlier. This is much earlier than reported for centipedes in previous surveys of germ cell development (Extavour and Akam, 2003; Nieuwkoop and Sutasurya, 1981), and resolves an ambiguity in the older literature (Heymons, 1901). Furthermore, we show that the *Strigamia* PGCs accumulate at the blastopore; that they become internalized during closure of the blastopore, independent of the hindgut invagination; and that they undergo a long-range migration to the embryonic gonad. This is the first definitive evidence for germ cells displaying these characteristics in any myriapod group.

It will be an interesting area for future work to examine molecular PGC markers in other myriapod groups, including other centipede orders, to corroborate (or otherwise) previous studies that used only cytological criteria to identify PGCs. This will clarify if morphologically indistinguishable PGCs also segregate early in the embryonic development of other myriapods, and if the position of segregation bears any relation to a blastopore structure. Only with this information will we be able to make correct phylogenetic inferences about the ancestral state of the PGCs in the Myriapoda, with broader implications for understanding the evolution of germ cells in insects, crustaceans and chelicerates.

### Implications for the mechanism of germ line specification in *Strigamia*

The precise mechanism of PGC specification remains unclear in *Strigamia*. During the early rounds of zygotic cell division at the centre of the egg, *nanos2* transcripts are expressed in a subpopulation of cleavage cells (Fig. 3C). This is suggestive evidence for asymmetric segregation of a germ plasm. However, we cannot rule out alternative hypotheses (such as variation in expression linked to position in the cell cycle) and so do not consider the evidence definitive.

We found no evidence for localization of *nanos2* or *vasa* transcript to a specialized region of cytoplasm in developing oocytes. However, we found a surprising localization of Vasa protein within the germinal vesicle of developing oocytes. In a range of taxa where Vasa localization has been examined during gametogenesis, Vasa protein is localized to the perinuclear cytoplasm, not within the nucleus itself (Gustafson and Wessel, 2010; Knaut et al., 2000; Liang et al., 1994; Raz, 2000). In some taxa with a cytoplasmic mode of specification, such as *Drosophila*, Vasa protein is also localized to the germ plasm at later stages of oogenesis (Liang et al., 1994), but again is not observed inside the oocyte nucleus.

In the grasshopper *Schistocerca americana*, it has been suggested that localization of maternal Hunchback protein in the germinal vesicle might act as a mechanism for localizing the protein in the early embryo (Patel et al., 2001). During *Schistocerca* oogenesis Hunchback protein is localized to and accumulates in the germinal vesicle. In further development the germinal vesicle moves to the posterior of the oocyte and, in the final stages of oogenesis, the Hunchback protein is released from the germinal vesicle into the surrounding cytoplasm. This leads to an asymmetric localization of maternal Hunchback protein in the posterior cytoplasm of the freshly laid egg, and it is suggested that this asymmetry determines the position of the embryonic primordium with respect to the long axis of the egg (Patel et al., 2001). It is possible that, through a similar mechanism, the germinal vesicle localization of maternal Vasa protein observed in *Strigamia* might be a way of achieving asymmetric localization of Vasa protein at the time of fertilization, and thus perhaps in early cleaving embryos. However, we do not have access to these stages in *Strigamia* and at present have not been able to visualize Vasa protein localization during embryogenesis. In all, we consider the mode of germ cell specification to be as yet undefined and a matter for future investigation.

### The *Strigamia* blastopore and its relation to the gut cavity

The mode of gastrulation is extremely variable across the arthropods (Anderson, 1973). Nevertheless a common structure, a blastopore (defined as a site of localized cell internalization during gastrulation), is recognized in the embryos of many different arthropods (Gerberding and Patel, 2004; Manton, 1949; Mittmann and Wolff, 2012; Wolff and Hilbrant, 2011). However, a blastopore is not necessarily the only site of cell internalization during gastrulation, and does not bear any necessary relation to the future gut cavity. Indeed arthropods display a range of relationships between the blastopore and the oral and anal openings of the gut (summarized in Martin-Duran et al., 2012).

Previous descriptions of the morphology of the *Strigamia* blastopore, observed through nuclear stains, showed that the blastopore is never an opening into an overt cavity (Brena and Akam, 2012). Rather, it is a discontinuity in the blastoderm epithelium that appears as an opening to the yolk, covered by a low-density cell population. Given this, it was not clear how the cavity of the hindgut formed, and if it bore any relation to the blastoporal opening. We find that the hindgut cavity is formed as a result of a new invagination within *brachyury*-expressing cells. It is clear that at least the posterior part of the blastopore remains open as the hindgut invagination forms (stage 3; Fig. 4), and does not close until later in development, at approximately the end of stage 4.1 (Fig. 5), when it closes over the germ cells, which lie posterior to and outside of the hindgut.

## Appendix A Supplementary materials

Supplementary data: **Fig. S1. Staging blastoderm embryos using an independent marker,*****sp5*****expression.** Relates to embryos in Fig. 4A and C in the main text. From stage 2.3 onwards, *Strigamia* embryos are relatively easy to stage based on the number of leg-bearing segments and other morphological criteria (Brena and Akam, 2012). However, the gross appearance of embryos changes little over stage 2.2. Therefore to put stage 2.2 embryos into developmental order requires an independent staging marker. In this case, we used the expression of another gene, the *Strigamia sp5* orthologue. *sp5* expression shows a clearly recognizable pattern of maturation over stage 2.2 (Vera Hunnekuhl unpublished data). In early stage 2.2, *sp5* is expressed as broad ring extending around the whole circumference of the egg with poorly defined borders. Over stage 2.2, the ventral part of the ring becomes increasingly sharp and narrows down. We used this pattern of maturation of *sp5* to confirm the developmental series of *bra1* expression over stage 2.2. Embryos were double stained to detect the expression of both genes at once; *bra1* was detected with Fast Red dye (Roche) and *sp5* was detected with BM purple dye (Roche). (A1)–(C1) *bra1* expression at the blastopore; same embryos as seen in Fig. 4A, C and (A2–C2) Fluorescent image of the Fast Red signal from *bra1* expression (red) and nuclear stain (blue). In ventral view only the anterior part of the blastoporal *bra1* expression is just visible at the posterior margin of the egg. Shadow of *sp5* expression is visible due to quenching of the nuclear stain by BM purple. (A3–C3) Bright field image showing the ventral part of the ring of *sp5* expression; note how it sharpens and narrows over time. (A4–C4) Overlay of fluorescent and bright field images to show the relative position of the *sp5* and *bra1* expression. Each row is accompanied by three adjacent panels. These are high magnification transverse sections in the approximate regions indicated by the boxed areas in (A4, B4, C4). Superficial cells are oriented to the top. The order of the boxed areas from top to bottom in column 4 corresponds to the order of the panels from top to bottom in column 5 (such that the top panel corresponds to the top boxed area etc.). These cross- sections shows that at least part of the posterior hemisphere of the blastoderm is already multi-layered by the time that the early, circular patch of *bra1* expression appears in (A1). All are ventral views, except (A1, B1, C1) that are posterior pole/blastopore views. White asterisks in (A2, C1) indicate artefactual tears. In the main images, scale bar is 100 μm. In panels, scale bar is 10 μm.
