# Supplementary figures and images for: Germ cells of the centipede Strigamia maritima are specified early in embryonic development
Source: Dev Biol. 2014 Aug 15;392(2):419–30. doi: 10.1016/j.ydbio.2014.06.003 (PMC4111900; doi:10.1016/j.ydbio.2014.06.003)

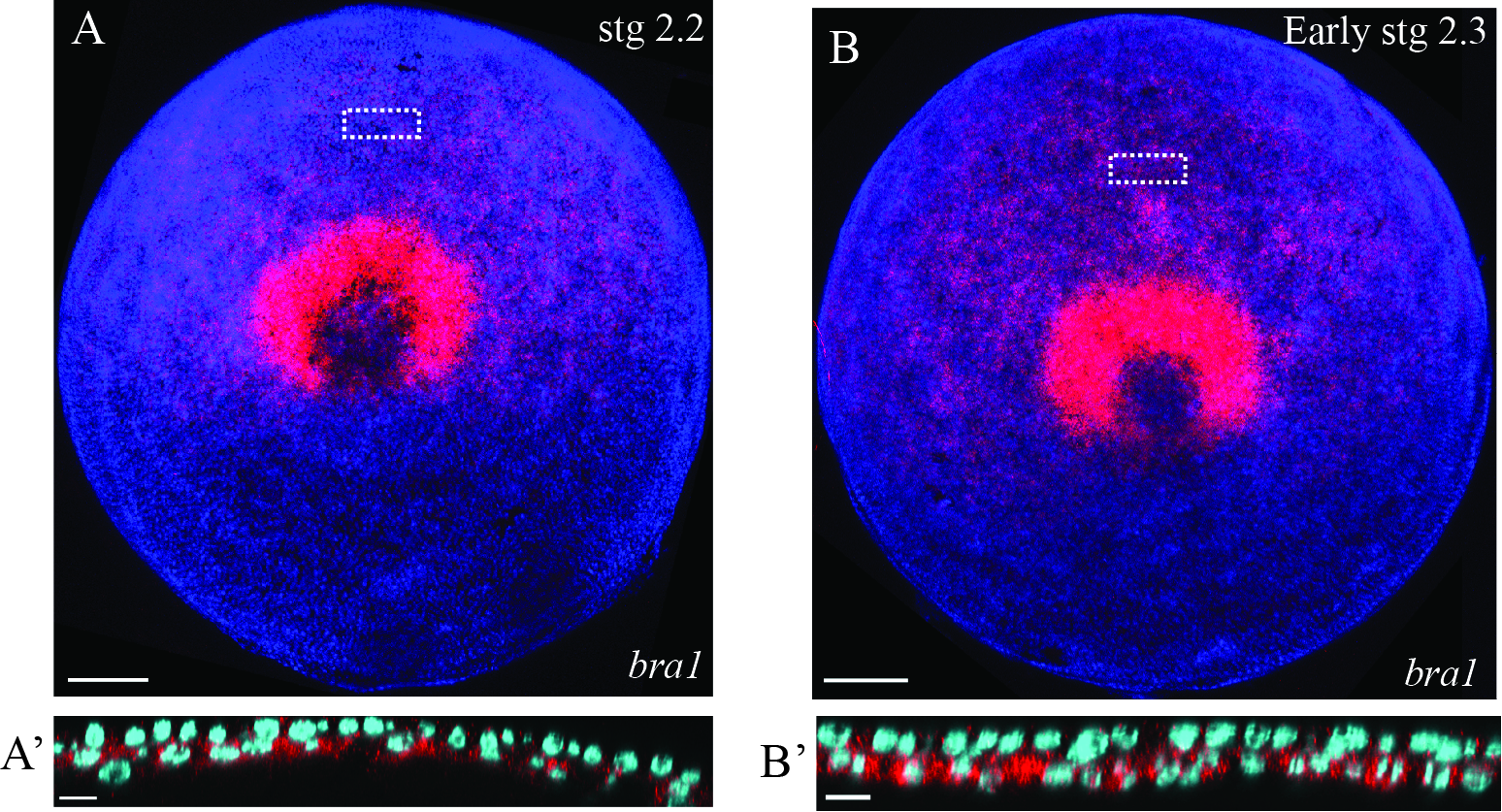

Supplement: Supplementary file 1 — Supplementary data: Fig. S1. Staging blastoderm embryos using an independent marker,sp5expression. Relates to embryos in Fig. 4A and C in the main text. From stage 2.3 onwards, Strigamia embryos are relatively easy to stage based on the number of leg-bearing segments and other morphological criteria (Brena and Akam, 2012). However, the gross appearance of embryos changes little over stage 2.2. Therefore to put stage 2.2 embryos into developmental order requires an independent staging marker. In this case, we used the expression of another gene, the Strigamia sp5 orthologue. sp5 expression shows a clearly recognizable pattern of maturation over stage 2.2 (Vera Hunnekuhl unpublished data). In early stage 2.2, sp5 is expressed as broad ring extending around the whole circumference of the egg with poorly defined borders. Over stage 2.2, the ventral part of the ring becomes increasingly sharp and narrows down. We used this pattern of maturation of sp5 to confirm the developmental series of bra1 expression over stage 2.2. Embryos were double stained to detect the expression of both genes at once; bra1 was detected with Fast Red dye (Roche) and sp5 was detected with BM purple dye (Roche). (A1)–(C1) bra1 expression at the blastopore; same embryos as seen in Fig. 4A, C and (A2–C2) Fluorescent image of the Fast Red signal from bra1 expression (red) and nuclear stain (blue). In ventral view only the anterior part of the blastoporal bra1 expression is just visible at the posterior margin of the egg. Shadow of sp5 expression is visible due to quenching of the nuclear stain by BM purple. (A3–C3) Bright field image showing the ventral part of the ring of sp5 expression; note how it sharpens and narrows over time. (A4–C4) Overlay of fluorescent and bright field images to show the relative position of the sp5 and bra1 expression. Each row is accompanied by three adjacent panels. These are high magnification transverse sections in the approximate regions indicated by the boxed areas [file mmc4.zip › Fig. S2.tif]
